# Double *In situ* Hybridization for MicroRNAs and mRNAs in Brain Tissues

**DOI:** 10.3389/fnmol.2016.00126

**Published:** 2016-11-22

**Authors:** Atsushi Kasai, Sora Kakihara, Hiroki Miura, Ryo Okada, Atsuko Hayata-Takano, Keisuke Hazama, Misaki Niu, Norihito Shintani, Takanobu Nakazawa, Hitoshi Hashimoto

**Affiliations:** ^1^Laboratory of Molecular Neuropharmacology, Graduate School of Pharmaceutical Sciences, Osaka UniversitySuita, Japan; ^2^Molecular Research Center for Children's Mental Development, United Graduate School of Child Development, Osaka University, Kanazawa University, Hamamatsu University School of Medicine, Chiba University and University of FukuiSuita, Japan; ^3^iPS Cell-based Research Project on Brain Neuropharmacology and Toxicology, Graduate School of Pharmaceutical Sciences, Osaka UniversitySuita, Japan; ^4^Department of Pharmacology, Graduate School of Dentistry, Osaka UniversitySuita, Japan; ^5^Division of Bioscience, Institute for Datability Science, Osaka UniversitySuita, Japan

**Keywords:** *in situ* hybridization, miRNA, parvalbumin, miR-34a, miR-181a, miR-9, miR-100, miR-219

## Abstract

MicroRNAs (miRNAs) participate in a variety of functions in the brain. Understanding the *in vivo* localization of miRNAs is an important step for uncovering their roles in brain function. However, the *in situ* detection of low-abundance miRNAs in brain tissues remains difficult and requires extensive optimization of *in situ* hybridization (ISH) protocols in individual laboratories. Thus, detailed information regarding experimental conditions would serve as a useful reference for researchers in this field. Here, we investigated and summarized the effects of adjusting a series of critical steps, including tissue fixation, probe accessibility and hybridization stringency, to standardize the currently used miRNA ISH procedures. As a result, we successfully detected several low-abundance miRNAs by ISH using the following experimental conditions: (1) use of fresh brain tissues, (2) digestion of brain samples with proteinase K, (3) LNA-probe hybridization at a temperature 37°C below the melting temperature of the RNA, (4) performance of high-stringency wash steps using 50% formamide in 1 × standard saline citrate (SSC) buffer. RT-PCR of the punched-out tissues using TaqMan^TM^ primers confirmed the ISH results. Finally, double-fluorescence ISH successfully demonstrated the colocalization of miRNAs and mRNAs. Thus, the detailed information regarding the miRNA ISH procedures used in this study may help to resolve the technical hurdles observed in the *in vivo* localization of miRNAs, and the elucidation of the specific roles of miRNAs.

## Introduction

MicroRNAs (miRNAs) serve as post-transcriptional fine-tuners of gene expression (Meister, 2007), and have become a focus of interest in both basic and translational neuroscience research (Soreq, 2014). A detailed spatial expression analysis of miRNAs in brain tissue is an important step to elucidate their functions. A powerful tool for studying miRNA localization is *in situ* hybridization (ISH), which uses 2′-O, 4′-C-methylene bridged nucleic acid/locked nucleic acid (LNA; Torigoe et al., 2001) probes that have an increased melting temperature (*T*_*m*_) and provide enhanced stringency with short probes (Kloosterman et al., 2006). Several miRNA ISH protocols have been reported (Thompson et al., 2007; Obernosterer et al., 2007; Jørgensen et al., 2010; Chaudhuri et al., 2013). Pena et al., reported *in situ* localization of 48 out of 126 miRNAs (Pena et al., 2009). ISH reliably detects high-abundance miRNAs, such as miR-124, in brain tissues (Sanuki et al., 2011), whereas detection of low-abundance miRNAs in brain tissues still proves difficult and requires extensive optimization in each laboratory. Therefore, this article provides detailed information regarding the experimental conditions tested in our laboratory, which may serve as a useful reference for researchers in this field.

The specificity and sensitivity of the ISH signals are highly dependent on the stringency of the hybridization, which is determined by the thermal stability of the probes and the concentration of formamide used in the hybridization and post-wash buffers (Obernosterer et al., 2007). In addition to these conditions, sample fixation, or probe accessibility can also influence the degree of signal intensity. The present study was designed to investigate the effects of a series of critical procedures, including the following: (1) tissue fixation, (2) proteinase K treatment, (3) hybridization temperature, and (4) stringent washes. Using this optimized protocol, we detected the ISH signals specific to several miRNAs, whose expression patterns are unknown. Moreover, the optimized miRNA ISH protocol can be used with mRNA ISH to identify cell-type specific expression of miRNAs. Thus, our study contributes to the field by enhancing the robustness of the miRNA ISH technique.

## Materials and equipment

### Animals

All animal care and handling procedures were performed according to the Guiding Principles for the Care and Use of Laboratory Animals, approved by the Animal Care and Use Committee of the Graduate School of Pharmaceutical Sciences, Osaka University, Japan. Male 7–8 week old C57BL/6J mice were purchased from Shimizu Laboratory Supplies (Kyoto, Japan). The mice were kept on a 12 h light/dark cycle (lights on at 8:00 a.m.) and in a controlled room (temperature 22–24°C). Food and water were available *ad libitum*.

### Equipment used for ISH

Cryostat (CM1520, Leica, Wetzlar, Germany).

Hybridization oven (HB-80, TAITEC, Saitama, Japan) and/or Hybridizer (S2450, DAKO Denmark A/S, Glostrup, Denmark).

Fluorescence microscope (BZ-9000, KEYENCE, Osaka, Japan).

### Buffers and solutions

Ensure that all equipment and working surfaces remain RNase-free by using RNase Away (Molecular BioProducts, San Diego, CA) or dry heat sterilization at 200°C for at least 5 h.

The following buffers or solutions can be prepared in advance.

1. *10*× *phosphate buffered saline (PBS), pH 7.38*
  30 g of NaCl.
  1 g of KCl.
  12 g of Na_2_HPO_4_•12H_2_O.
  1 g of KH_2_PO_4._
  Make up to 500 mL with ultrapure water which has been RNase-inactivated by diethyl pyrocarbonate (DEPC) treatment.
  Store at room temperature.

Note: DEPC treatment

Add 1 mL of DEPC per 1 L of solution (0.1% v/v).
  Incubate at room temperature for 1 h and autoclave at 121°C for 20 min.
2. *12% paraformaldehyde (PFA)*
  60 g of PFA.
  Make up to 500 mL with 1 × PBS.
  Store at −20°C and protect from light.
  Dilute 12% PFA with PBS to 4% PFA on the day of the experiment.
  Store in aliquots at −30°C.
3. *20* × *standard saline citrate (SSC), pH 7.0*
  175 g of NaCl.
  88.2 g of trisodium citrate dehydrate.
  Make up to 1000 mL with ultrapure water.
  Adjust pH with 1 M HCl and treat the solution with DEPC.
  Store at room temperature.
4. *Hybridization buffer*
  25 mL of deionized formamide.
  0.5 mL of 1 M Tris-HCl, pH 7.2.
  5 mL of 20 × SSC.
  1 mg/mL of yeast tRNA.
  10 mg of sodium dodecyl sulfate (SDS).
  10 mL of 50% dextran sulfate.
  1 mL of 50 × Denhardt's solution.
  Make up to 50 mL with ultrapure water.
  Store in aliquots at −20°C.
5. *10*× *Tris-HCl, NaCl (TN) buffer, pH7.3*
  13.4 g of Trizma base.
  140 g of Trizma hydrochloride.
  87.7 g of NaCl.
  Make up to 1000 mL with ultrapure water.
  Store at room temperature.
6. *10*× *maleic acid buffer, pH 7.5*
  58.1 g of maleic acid.
  43.8 g of NaCl.
  Adjust pH using NaOH.
  Make up to 500 mL with ultrapure water.
  Store at room temperature.
7. *1 M Tris-HCl, pH 9.5*
  Dissolve 121 g of Trizma base in 900 mL of ultrapure water.
  Adjust pH using 12 M HCl.
  Store at room temperature.
8. *10*× *PBS, pH 5.8*
  40.0 g of NaCl.
  1.0 g of KCl.
  13.6 g of Na_2_HPO_4_•12H_2_O.
  42.3 g of NaH_2_PO_4_•2H_2_O.
  Make up to 500 mL with ultrapure water.
  Store at room temperature.
9. *0.2 M phosphate buffer, pH 7.0*
  4.4 g of Na_2_HPO_4_•12H_2_O.
  1.2 g of NaH_2_PO_4_•2H_2_O.
  Make up to 100 mL with ultrapure water.
  Store at room temperature.
10. *Glycerol gelatin*
  25 mL of 0.2 M phosphate buffer.
  50 mg of sodium azide.
  25 mL of glycerol.
  3.8 g of gelatin.
  Mix while heating to 80°C and store at 4°C.
11. *Tris-NaCl-Tween (TNT) buffer (0.05% Tween-20 in TN buffer)*
  Dilute 0.5 mL of Tween-20 in 1000 mL of TN buffer.
  Store at room temperature.
  N.B. The following buffers must be prepared immediately prior to use.
12. *Acetylation solution*
  2.4 g of triethanolamine.
  1.4 g of NaCl.
  Make up to 160 mL with ultrapure water.
  Add 400 μL of acetic anhydride just before dipping the slides.
13. *1% blocking buffer*
  Dissolve 1 g of Blocking Reagent (Roche Diagnostics, Mannheim, Germany, cat.no. 11096176001) in 100 mL of TN buffer while heating.
14. *Alkaline phosphatase reaction buffer*
  100 mL of 1 mM Tris-HCl, pH 9.5.
  5.9 g of NaCl.
  4.8 g of MgCl_2._
  Make up to 1000 mL with ultrapure water.
15. *Nitro blue tetrazolium (NBT)/5-bromo-4-chloro-3-indolyl-phosphate (BCIP) in alkaline phosphatase reaction buffer*
  5 μL of NBT.
  3.75 μL of BCIP.
  Add 1 mL of alkaline phosphatase reaction buffer.
16. *Fluorophore amplification reagent working solution*
  Dilute the fluorophore amplification reagent stock solution from the tyramide signal amplification (TSA) Plus fluorescein kit 1:50 with the 1 × plus amplification diluent, also from the kit (PerkinElmer, Waltham, MA).
  Add the resulting solution to an equal amount of ultrapure water.
17. *Biotin amplification reagent working solution*
  Dilute the biotin amplification reagent stock solution from the TSA Plus biotin kit 1:50 with the 1 × plus amplification diluent from the same kit (PerkinElmer).

### Stepwise procedures

#### Tissue preparation

1. For fresh samples, brains were removed from the skulls of adult mice (8–9 week old) and rapidly frozen by immersion in isopentane that had been chilled on dry ice.
2. For fixed samples, the mice were anesthetized with sodium pentobarbital (50 mg/kg, intraperitoneal injection), and perfused through the left ventricle with 4% PFA. The brains were removed, postfixed overnight at 4°C in 4% PFA, and cryoprotected using 20% sucrose in PBS.
3. The brain samples were cut at a thickness of 20 μm on a cryostat (CM1520, Leica, Wetzlar, Germany) and collected on Matsunami Adhesive Silane (MAS)-coated glass slides (Matsunami Glass Ind., Ltd, Osaka, Japan). Coronal sections (−1.82 mm from bregma) containing the hippocampus (Hip), the thalamic reticular nucleus (TRN), the amygdala (Am), the choroid plexus of the ventricles (ChP), and the central medial nucleus of the thalamus (CM) were obtained.

#### RNA probe synthesis

1. The fluorescein-labeled probe for parvalbumin (PV) (Puig et al., 2010) was prepared by transcribing a *Hin*dIII-linearized plasmid using T7 RNA polymerase, Fluorescein RNA Labeling Mix, Protector RNase Inhibitor, and DNase I recombinant (Roche Diagnostics).
2. The RNA probes were purified using Illustra ProbeQuant G-50 Micro Columns (GE Healthcare Japan, Tokyo, Japan).

#### Semi-quantitative reverse-transcription (RT)-PCR

1. A brain sample from a particular brain region was punched out from a fresh brain section using a disposable biopsy puncher (φ1 mm) (Kai Medical, Gifu Japan). The punched-out brain regions were confirmed by cresyl violet/Nissl staining (Türeyen et al., 2004).
2. Total RNA extraction from brain samples was performed using an miRNeasy Mini Kit (QIAGEN, Hilden, Germany) according to the manufacturer's instructions.
3. Total RNA (10 ng for miRNA, 200 ng for mRNA) was reverse transcribed using a TaqMan™ MicroRNA Reverse Transcription Kit (Applied Biosystems, Foster City, CA) for miRNA or a Moloney murine leukemia virus reverse transcriptase (Invitrogen, Carlsbad, CA) for mRNA.
4. Real-time RT-PCR was performed according to the manufacturer's instructions using either TaqMan™ Universal Master Mix II (Applied Biosystems) with miRNA-specific TaqMan™ MicroRNA Assays (Applied Biosystems, snoRNA: Assay ID 001232; miR-34a: Assay ID 000426; miR-34c: Assay ID 000428), or GoTaq qPCR Master Mix (Promega, Madison, WI) with gene-specific primer sets [mouse glutamate decarboxylase 1 (Gad1): 5′-CCT TCG CCT GCA ACC TCC TCG AAC-3′ and 5′-GCG CAG TTT GCT CCT CCC CGT TCT T-3′ or mouse β-actin: 5′-TCC CAC ACT GTG CCC ATC TA-3′ and 5′-GCC ACA GGA TTC CAT ACC CA-3′].

### *In situ* hybridization (outlined as a flowchart in Figure 1)

#### Day 1

1. Desiccate sections for 10 min at 50°C.
2. Fix the sections with 4% PFA for 15 min at room temperature, then wash twice with PBS for 5 min.
3. Add 1 mL of proteinase K (indicated concentration) in PBS to the section in a humidified chamber, incubate for 30 min at 37°C, then incubate in 4% PFA for 15 min at room temperature, and finally rinse in distilled water.
4. Acetylate the sections in acetylation buffer for 10 min at room temperature, then wash with PBS for 5 min at room temperature.
5. Add 100 μL of hybridization buffer to each tissue section and cover the section in the hybridization chamber with a small piece of parafilm. Incubate for 30 min at the indicated temperature.
6. For TSA detection, add 1 pmol 5′ DIG-labeled LNA probe and 90 ng/kb fluorescein-labeled antisense PV RNA probe to 100 μL hybridization buffer, heat for 5 min at 65°C to denature the probe, and then immediately chill on ice for 5 min. For NBT/BCIP detection, add 1 pmol 5′ digoxigenin (DIG)-labeled LNA probe to 100 μL hybridization buffer, heat for 5 min at 65°C to denature the probe, and then immediately chill on ice for 5 min.
7. Replace the prehybridization buffer with the hybridization buffer containing the probe and allow hybridization to take place overnight at the temperatures calculated from the *T*_*m*_-values as indicated.

**Figure 1:**
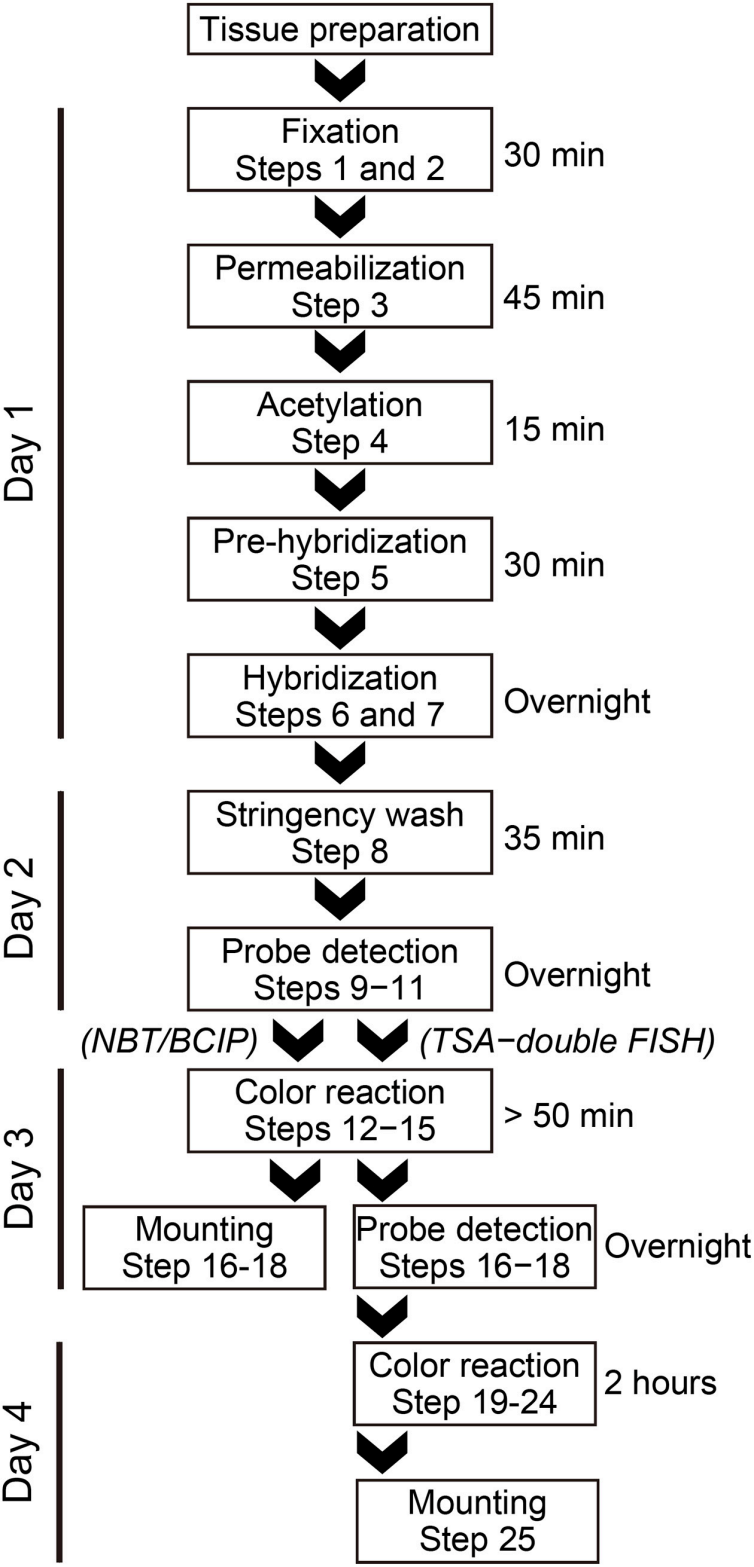
**A flow chart of the miRNA *in situ* hybridization procedure**.

#### Day 2

8. Wash sections twice with 1 × SSC containing the indicated concentrations of formamide for 10 min at the specified hybridization temperature. Then wash sections with 0.1 × SSC for 10 min at the specified hybridization temperature and subsequently with TN buffer for 5 min at room temperature.
9. Quench the endogenous peroxidase activity by incubating the sections in 3% hydrogen peroxide for 10 min, followed by washing three times in TN buffer for 3 min. This process is used with TSA detection.
10. Incubate sections in 1% blocking buffer for 1 h at room temperature.
11. Incubate sections overnight with alkaline phosphatase-labeled anti-DIG antibody (1:1000, Roche Diagnostics, for NBT/BCIP detection), with peroxidase-labeled anti-fluorescein antibody (1:20, Roche Diagnostics, for double fluorescent ISH (FISH)-TSA detection in 1% blocking buffer at 4°C.

### For double FISH using TSA detection

#### Day 3

12. Wash the sections three times with TNT buffer for 5 min at room temperature.
13. Wash the sections with 1 × plus amplification diluent from the TSA Plus Fluorescein kit.
14. Pipette 100 μL fluorophore amplification reagent working solution onto each tissue section and incubate for 20 min at room temperature.
15. Wash the sections three times with TNT buffer for 5 min at room temperature on a shaker.
16. Quench the endogenous peroxidase activity by incubating the sections in 3% hydrogen peroxide for 10 min, followed by washing three times with TN buffer for 3 min.
17. Incubate sections in 1% blocking buffer for 1 h at room temperature.
18. Incubate sections overnight with peroxidase-labeled anti-DIG antibody (1:250; Roche Diagnostics) in 1% blocking buffer at 4°C.

#### Day 4

19. Wash the sections three times with TNT buffer for 5 min at room temperature.
20. Wash the sections with 50 μL amplification diluent from the TSA Plus biotin kit.
21. Pipette 100 μL biotin amplification reagent working solution onto each tissue section and incubate for 10 min at room temperature.
22. Wash the sections three times with TNT buffer for 5 min at room temperature with gentle shaking.
23. Pipette 100 μL of Texas Red Streptavidin (3:100) and if necessary, 10 μg/mL Hoechst 33258 in TNT buffer onto each tissue section and incubate 1 h at room temperature.
24. Wash the sections three times with TNT buffer for 5 min at room temperature with gentle shaking.
25. Mount the sections on slides with Fluoromount (Diagnostic BioSystems, Pleasanton, CA, cat. no. K024).
26. Take images of sections using a fluorescence microscope (Keyence filter: GFP, Ex 470/40, DM495, Em 525/50; TxRed, Ex 560/40, DM 595, Em 630/60).

### For NBT/BCIP detection

#### Day 3

12. Wash sections with 0.3% Tween 20 in maleic acid buffer four times for 10 min.
13. Wash sections twice with alkaline phosphatase reaction buffer for 5 min at room temperature.
14. Incubate sections in NBT/BCIP solution in alkaline phosphatase reaction buffer for 1 h or overnight in a slide mailer (AS ONE Corporation, Osaka, Japan) protected from light.
15. Wash sections with PBS, pH 5.8 for 10 min at room temperature.
  If necessary, stain nuclei with 10 μg/mL hoechst 33258 in PBS and wash twice with PBS for 5 min.
16. For long-term storage of stained samples, incubate sections in 4% PFA for 10 min at room temperature.
17. Mount sections with glycerol gelatin and seal the slides with nail polish.
18. Capture images using a fluorescence microscope.

## Anticipated results

**Figure 2:**
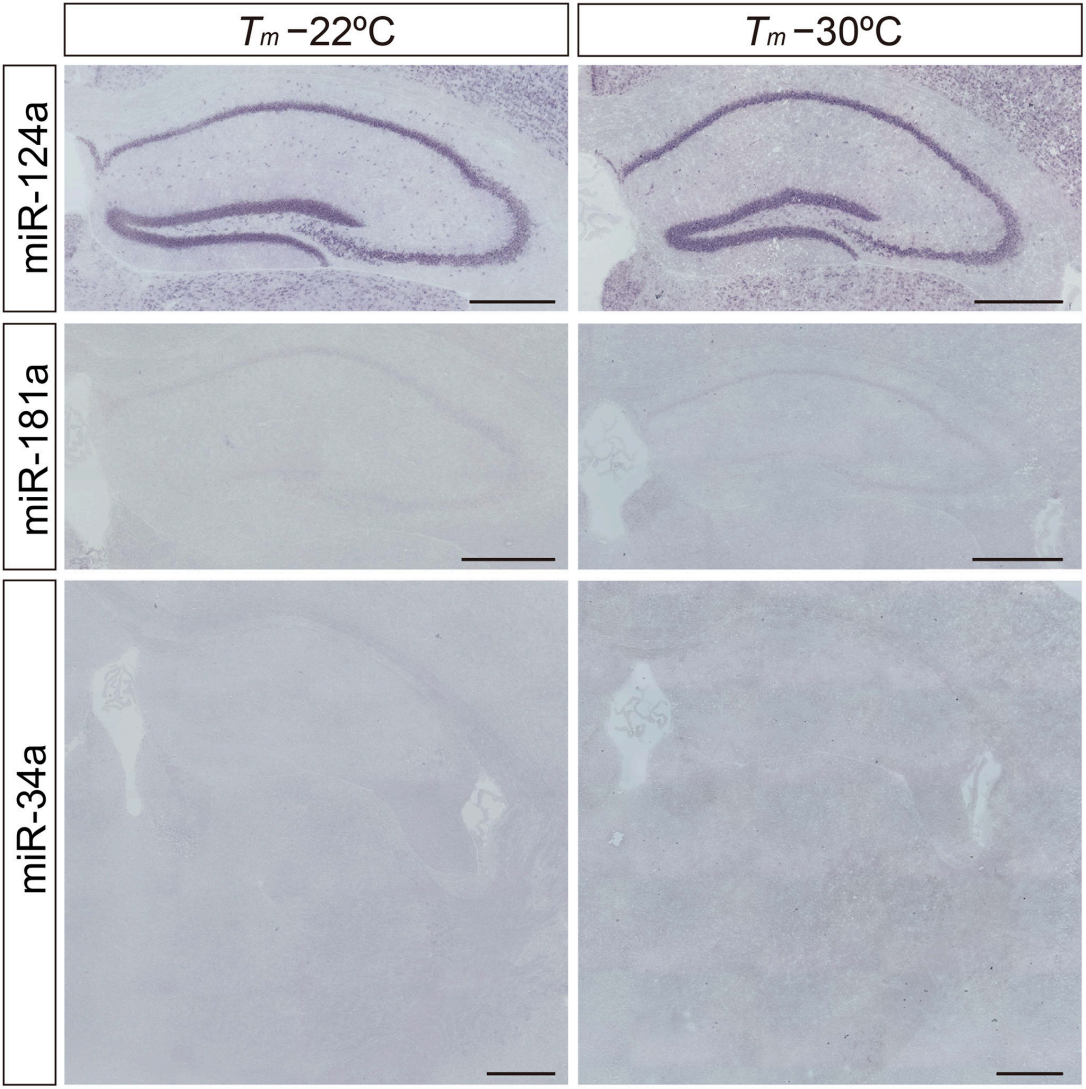
**Limitation of the *in situ* detection of miRNA in mouse brain tissues**. miRNA ISH reported by Silahtaroglu et al. (2007) was performed with commercially available LNA-modified probes on adult mouse brain sections including the Hip and TRN at 22° and 30°C below each *T*_*m*_-value. miR-124a (upper panels) was used as an example of a highly expressed miRNA in the brain. miR-181a (middle panels) and miR-34a (lower panels) were used as examples of low abundance miRNAs in the brain. Detection was carried out using the NBT/BCIP colorimetric method. Scale bars, 500 μm.

Neuron-specific miR-124a is useful for optimizing the detection sensitivity and performance of the miRNA ISH method in brain tissues, since miR-124a is expressed in abundance in the central nervous system (Deo et al., 2006). Consistent with previous studies (Silahtaroglu et al., 2007; Sanuki et al., 2011), the expression of miR-124a was clearly detectable in the brain using the protocol of Silahtaroglu et al. with NBT/BCIP detection. The miR-124a signal was detectable at hybridization temperatures of both 22° and 30°C below its RNA *T*_*m*_ in the Hip of the mouse brain (Figure 2). Next, we tested LNA probes against miR-181a and miR-34a using the same procedure as for the detection of miR-124a. However, miR-181a and miR-34a ISH signals were barely detectable when the hybridization temperature was set 22° or 30°C below the *T*_*m*_ of the miRCURY™ LNA detection probes (Figure 2). MiRNA profiles of small RNA cDNA libraries from size-fractionated total brain RNA show that clone counts of miR-181a and miR-34a are one-tenth and one-thirtieth of the miR-124a clone counts, respectively (Pena et al., 2009). In addition, we performed TaqMan™ RT-PCR and showed that the relative expression levels of miR-181a and miR-34a in the hippocampus were one-half and one-eighth of those of miR-124a, respectively. These data indicate that fine-tuning of the miRNA ISH protocol is required, especially for the detection of low-abundance miRNAs.

**Figure 3:**
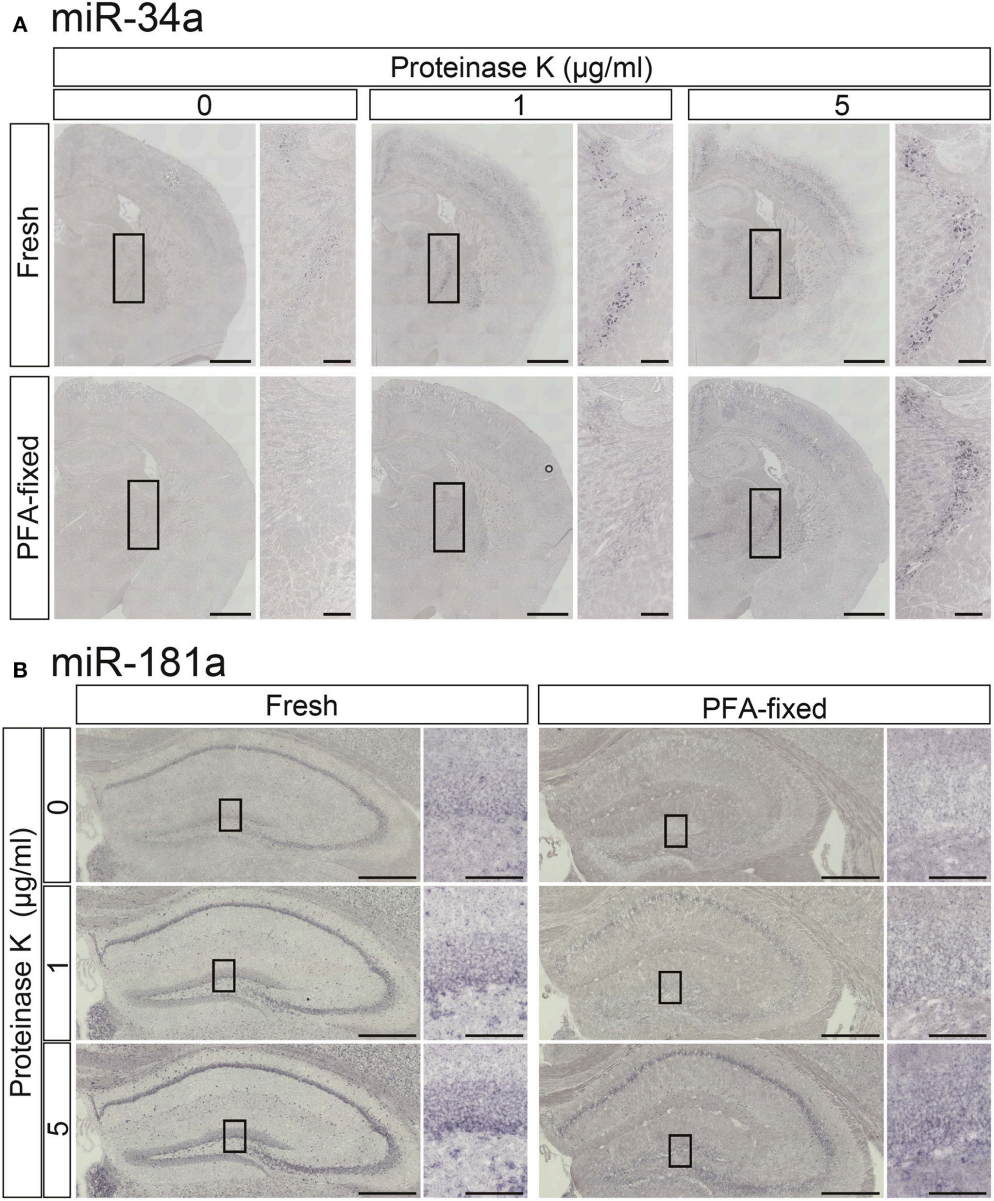
**Comparisons of the signal intensities of miRNA ISH in fresh and fixed brain sections with or without proteinase K digestion**. *In situ* detection of miR-34a **(A)** and miR-181a **(B)** was performed in adult mouse brain sections. The hybridization temperature was 37°C below the *T*_*m*_ of each RNA. Detection was carried out using the NBT/BCIP colorimetric method. The sections were treated with or without proteinase K at the indicated concentrations. **(A)** Representative images of miR-34a in fresh (upper panels) and PFA perfusion-fixed (lower panels) brain sections are shown. The insert images are a higher magnification of the boxed brain sections including the TRN. Scale bars in **(A)**, 1 mm; 0.2 mm in the inserted images. **(B)** ISH signals for miR-181a in fresh (left panels) and PFA perfusion-fixed (right panels) brain sections are shown. The insert images are a higher magnification of the boxed brain sections including the hippocampal DG **(B)**. Scale bars in **(B)**, 500 μm; 100 μm in the inserted images.

To increase the detection sensitivity of miRNA ISH we first tested the influence of proteinase K digestion in PFA-perfused fixed and fresh-frozen brain samples using LNA-modified probes for miR-181a and miR-34a. In the TRN, which is comprised of a thin layer of gamma-aminobutyric acid (GABA) neurons surrounding the thalamus (Ohira et al., 2013), miR-34a was weakly detected in fresh samples but not in PFA-fixed samples without proteinase K treatment (Figure 3A). Proteinase K treatment clearly enhanced the miR-34a ISH signals in both fresh and PFA-fixed samples, although over-digestion, through the use of 5 μg/mL proteinase K, was evident at the edges of fresh brain samples (Figure 3A). The miR-181a signal in the Hip of fresh samples was more clearly visible when compared to the PFA-fixed samples (Figure 3B). As with the miR-34a signal, the intensity of the miR-181a signal was enhanced by proteinase digestion (Figure 3B). Tissue fixation prior to sectioning stabilized the tissue against over-digestion by proteinase K but altered the expression pattern of miR-181a in the hippocampal dentate gyrus (DG). In the fresh sample, the miR-181a signal was clear in the subgranular zone-granule cell layer of the hippocampal DG. In contrast, the miR-181a signal in the PFA-fixed sample was obscure in the subgranular zone-granule cell layer (Figure 3B). These data suggest that proteinase digestion was effective for detecting the ISH signal of low-abundance miRNAs in both fixed and fresh brain samples, and that 4% PFA perfusion and/or overnight fixation prior to sectioning of brain samples has the potential to influence both the intensity and pattern of miRNA ISH signals. Therefore, optimizing these parameters individually is essential for obtaining reproducible results and detecting miRNAs with high sensitivity.

**Figure 4:**
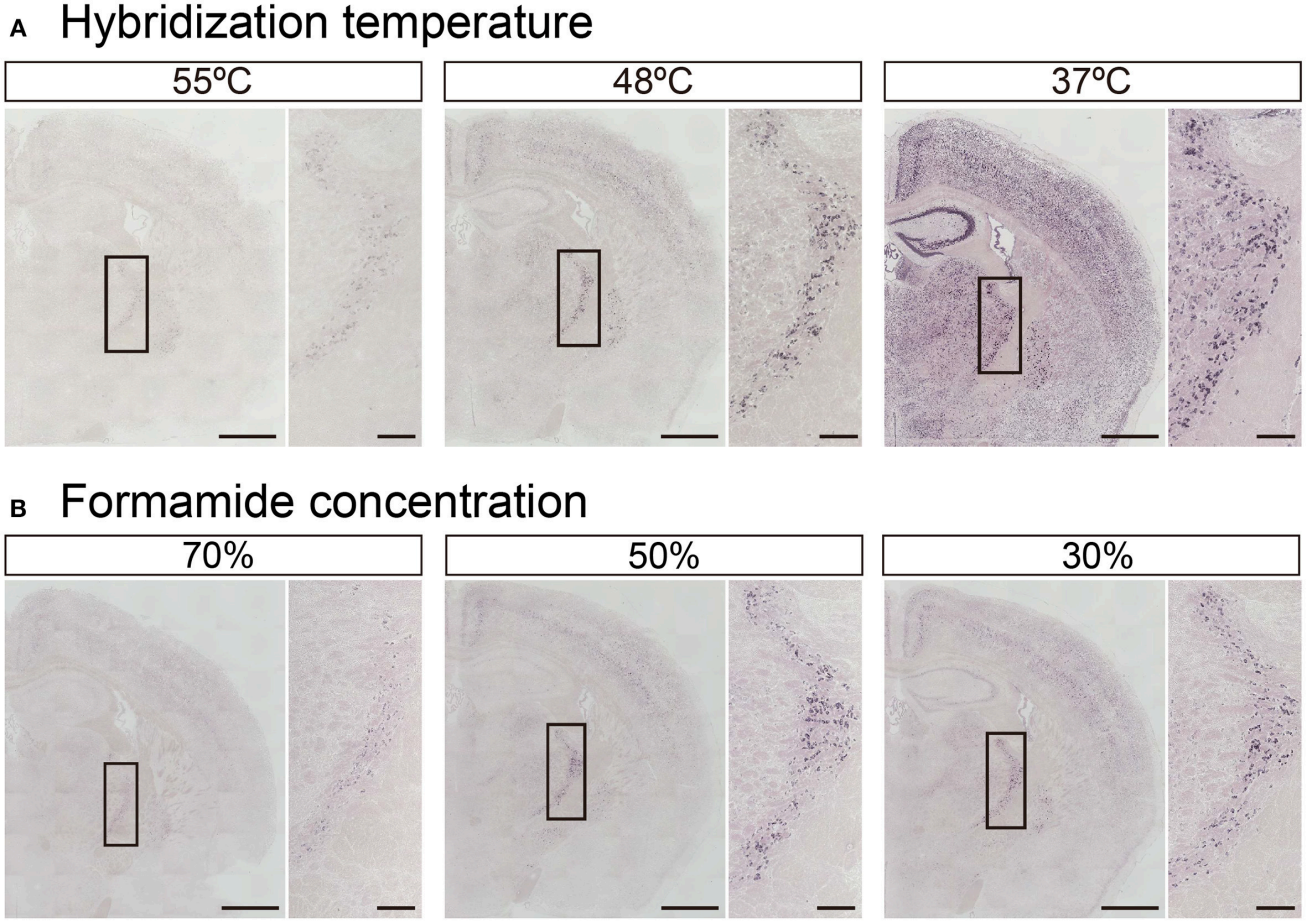
**Adjustment of hybridization stringency for miR-34a in fresh brain tissue**. ISH of miR-34a was performed in fresh samples treated with 1 μg/mL proteinase K. **(A)** miR-34a probes were hybridized at the indicated temperatures (55°, 48°, and 37°C) followed by washing with 50% formamide in 1 × SSC at the hybridization temperature. **(B)** The sections hybridized at 48°C were washed with buffer solution including the indicated percentages of formamide (70, 50, and 30%) in 1 × SSC. The insert images are a higher magnification of the boxed brain sections including the TRN. Scale bars, 1 mm; 0.2 mm in the magnified image.

In addition to tissue fixation and proteinase digestion, hybridization temperature, and stringent wash conditions also dictate the intensity and specificity of ISH signals. While Exiqon, the manufacturer of the miRCURY™ LNA probes, recommends hybridization at 30°C below each RNA *T*_*m*_ shown in Table 1, experimental *T*_*m*_-values of LNA-modified ISH probes often do not match the Exiqon-predicted *T*_*m*_-values. In fact, among the 148 probes examined, only 46 probes were identified as having an experimental *T*_*m*_-value within 1°C of the putative *T*_*m*_-value (Pena et al., 2009). The difference between predicted and experimental *T*_*m*_-values ranged from −12° to 16.5°C. Therefore, we examined how hybridization temperature affects the hybridization signals using LNA probes against miR-34a, whose predicted *T*_*m*_ is 85°C (Table 1). The miR-34a signal was evident when the probes were hybridized at 37° and 48°C, but the signal was significantly decreased at 55°C (Figure 4A). When the hybridization temperature was 48°C, the miR-34a signal was detected mainly in the TRN. When the hybridization temperature was 37°C, the signal intensities for miR-34a in the hippocampal CA and DG regions and ChP were comparable to those in the TRN (Figure 4A). These data imply that a hybridization temperature of 37°C is too low to specifically detect the expression of miR-34a. Next, since formamide is widely used as an organic solvent for ISH and can denature RNA (lowering *T*_*m*_-values), the impact of the formamide concentration in the stringency wash buffer on signal intensity was examined. As expected, the signal intensity for miR-34a increased in an inversely proportional manner to the formamide concentration (Figure 4B). The miR-34a signal in the TRN was evident when a 50% formamide solution was used (Figure 4B). In the Hip, a faint, non-specific ISH signal was detected when a 30% formamide solution was used (Figure 4B). These results suggest that stringent washing with a 50% formamide solution is most suitable for detecting the signal specific to miR-34a.

**Table 1:** **List of LNA probes and RNA *T*_*m*_-values for miRNA**.

**miRNA name**	**Probe sequence (5′ to 3′)**	**Exiqon RNA *T*_*m*_ (°C)**	**Product no. in Exiqon**
miR-124a	5DigN/GGCATTCACCGCGTGCCTTA	90	88066-01
miR-181a	5DigN/ACTCACCGACAGCGTTGAATGTT	85	18066-01
miR-34a	5DigN/ACAACCAGCTAAGACACTGCCA	85	38487-01
miR-34c	5DigN/GCAATCAGCTAACTACACTGCCT	86	38542-01
miR-9	5DigN/TCATACAGCTAGATAACCAAAGA	78	18198-01
miR-100	5DigN/CACAAGTTCGGATCTACGGGTT	83	18009-01
miR-219	5DigN/AGAATTGCGTTTGGACAATCA	78	38497-01

**Figure 5:**
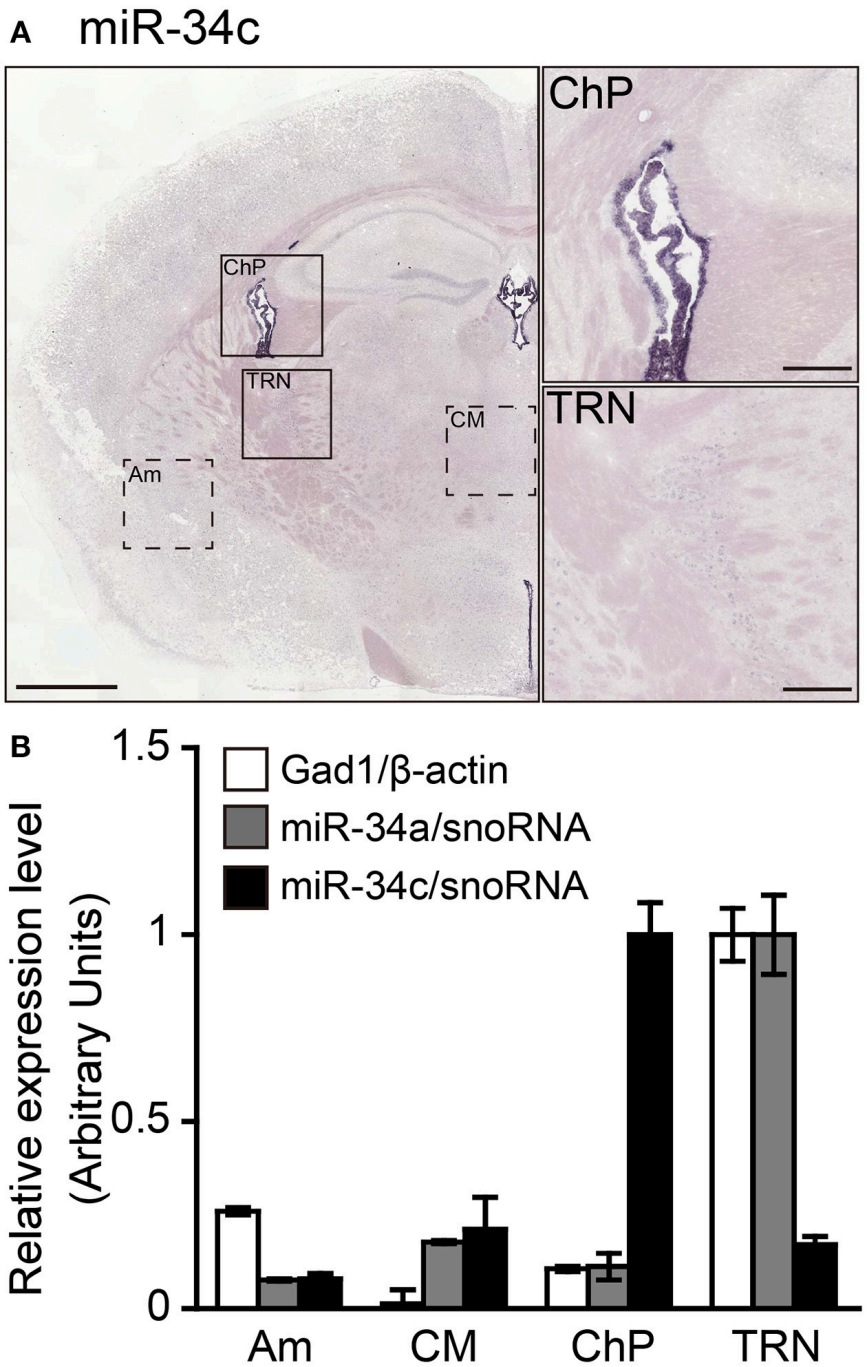
**Validation of the specificity of the ISH signals detected**. Sections including the TRN were hybridized using LNA-modified probes against miR-34c to test the specificity of the miR-34a ISH signal. ISH of miR-34c was performed in fresh samples treated with 1 μg/mL proteinase K. miR-34c probes were hybridized at 37° or 49°C below the *T*_*m*_-value, followed by washing with 50% formamide in 1 × SSC. **(A)** ISH signal of miR-34c is shown. The brain regions including the ChP or TRN in **(A)** are magnified in the right-hand panels. Scale bars, 1 mm; 0.2 mm in magnified images. **(B)** Relative expression levels of Gad1 (white bars), miR-34a (gray bars), and miR-34c (black bars) are shown in the brain regions, including the Am, CM, ChP, or TRN, which are shown as the dotted boxed areas or boxed areas in **(A)**. The expression levels of mRNA and miRNA were normalized to β-actin and snoRNA, respectively. Data are presented as the means ± SEM of three brains.

The miR-34a expression observed in the TRN does not exclude the possibility that the detected ISH signals may be excessive false positive signals due to detection under low-stringency conditions. To confirm the specificity of the miR-34a signal, we examined the differences in the expression of miR-34 family members (miR-34a/miR-34c, a/g caa c/t cagctaa g/ct acactgcc a/t) using ISH and TaqMan™ RT-PCR. In contrast to the miR-34a signal, Figure 4 shows that miR-34c signals were predominantly detected in the ChP at 49°C, a temperature 37°C below its *T*_*m*_ (Figure 5A). Next, we performed TaqMan™ RT-PCR to assess the expression of miR-34a/c in the punched-out tissues, including the ChP, the TRN, the Am, and the CM. According to the ISH results, the expression of miR-34a was highest in the TRN (Figure 5B). The miR-34a expression pattern was similar to that of Gad1, which is an enzyme implicated in GABA synthesis and a marker for GABAergic neurons. Moreover, miR-34c was expressed at high levels in the ChP (Figure 5B). The threshold cycle values of miR-34a and miR-34c in the TRN were 25.88 ± 0.10 and 30.81 ± 0.03, respectively (*n* = 3, average ± standard error). These data suggest that miRNA ISH signals are largely reliable up to 37°C below the *T*_*m*_, and that hybridization at 48°C below the *T*_*m*_ can result in false positive signals. Based on these observations, the hybridization temperature should be optimized for each individual ISH probe.

**Figure 6:**
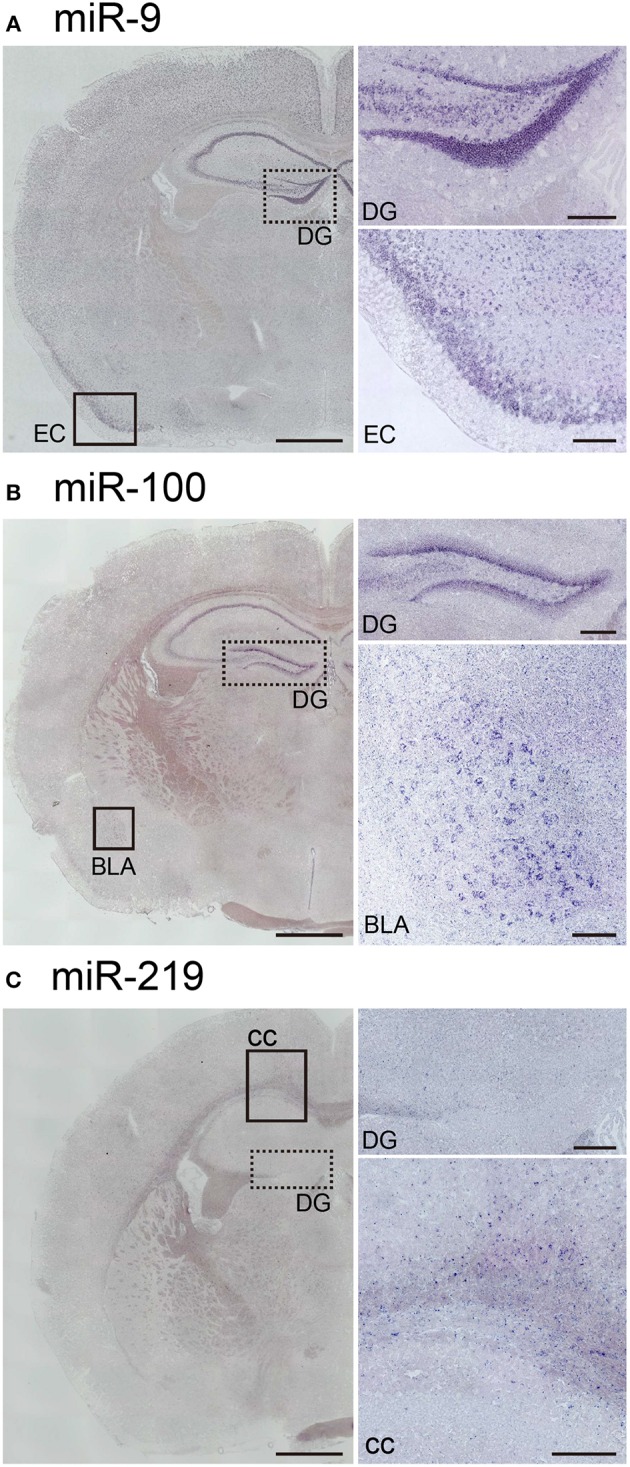
***In situ* localization of miR-9, miR-100, and miR-219 in the brain**. ISH of miR-9, miR-100, and miR-219 was performed in fresh samples treated with 1 μg/mL proteinase K. These probes were hybridized at 37°C below each *T*_*m*_-value, followed by washing with 50% formamide in 1 × SSC. **(A)** Representative images show the ISH signal for miR-9 in the brain. The higher magnification images correspond to the dotted-line and the black rectangle in **(A)**, the areas including the hippocampal DG and the entorhinal cortex (EC), respectively. **(B)** Representative images showing the ISH signal for miR-100 in the brain. The higher magnification images correspond to the dotted-line and the black rectangle in **(B)**, the areas including the hippocampal DG and the basolateral amygdala (BLA), respectively. **(C)** Representative images show the ISH signal for miR-219 in the brain. The higher magnification images correspond to the dotted line and the black rectangle in **(C)**, the areas including the hippocampal DG and both the hippocampal CA1 and the corpus callosum (cc), respectively. Scale bars, 1 mm; 0.2 mm in the magnified images.

Next, to assess the robustness of this protocol for performing miRNA ISH, we endeavored to detect the expression of three other miRNAs, namely miR-9, miR-100, and miR-219. MiR-9 is one of the most abundantly expressed miRNAs in the adult brain (Malmevik et al., 2016). MiR-9 has been also been detected in the piriform cortex and the hippocampal CA and DG regions in the adult mouse brain (Figure 6A). In addition, we examined the localization of miR-100 and miR-219, which both exhibit expression levels in the brain similar to that of miR-34a (Pena et al., 2009). MiR-100 was strongly expressed in the subgranular zone of the DG, the hippocampal CA1 and the basolateral amygdala (Figure 6B). Since miR-100 is upregulated in the Am by acute restraint stress, along with miR-34a and miR-34c (Haramati et al., 2011), the expression pattern of miR-100 might contribute to our understanding of its biological function. Moreover, we showed that miR-219 was expressed in abundance in the corpus callosum, but not in hippocampal neurons (Figure 6C). The expression pattern also corresponded to the function of miR-219, which regulates oligodendrocyte differentiation (Dugas et al., 2010; Zhao et al., 2010). Thus, each miRNA signal showed unique expression patterns in the brain, indicating that our protocol could be applied to the *in situ* detection of miRNAs of relatively low abundance in the brain.

**Figure 7:**
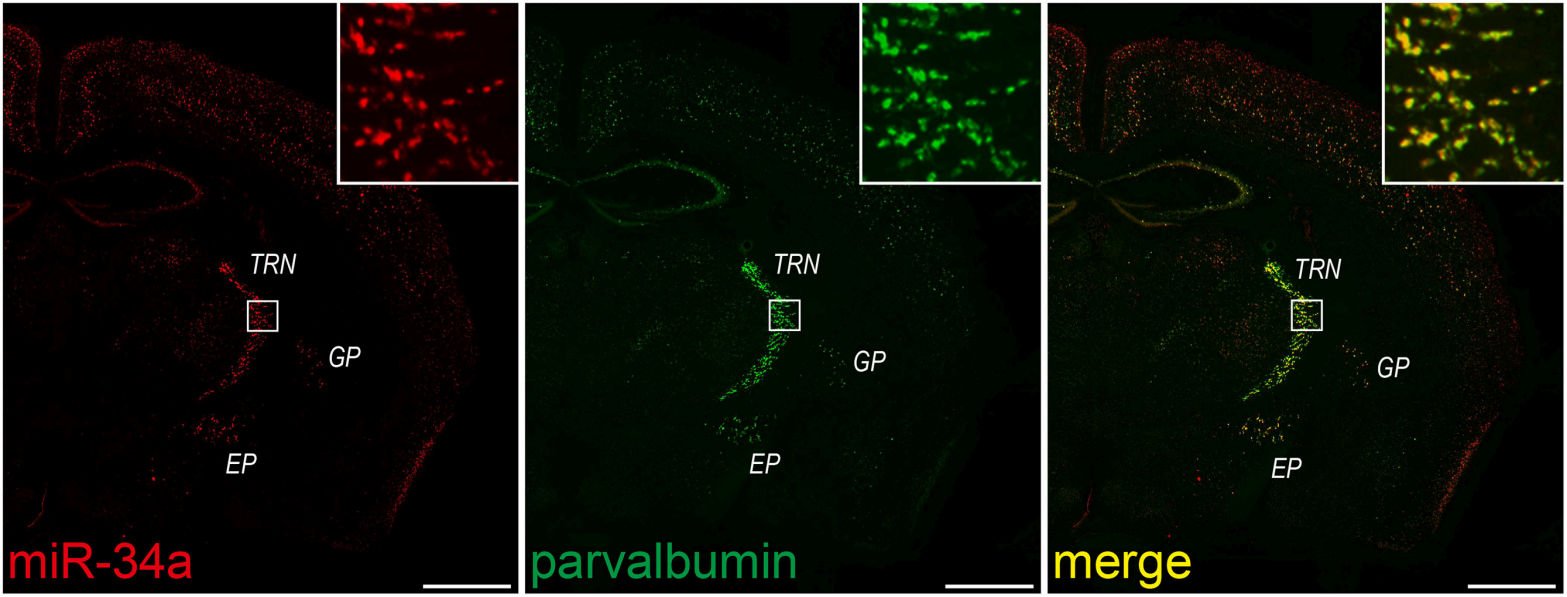
**miR-34a expression in parvalbumin-positive neurons in the TRN and cortical layer V**. Double FISH was performed using miR-34a and parvalbumin (PV) probes. DIG-labeled LNA-modified probes for miR-34a and fluorescein-labeled RNA probes for PV were hybridized at 48°C. Detection was carried out using the TSA Plus fluorescent kit for PV (green), followed by the TSA Plus biotin kit and Texas red streptavidin for miR-34a (red). The insert images are a higher magnification of the boxed brain areas including the TRN. EP, entopeduncular nucleus; GP, globus pallidus. Scale bars, 1 mm.

Double FISH is useful for identifying cells expressing a particular miRNA. Therefore, we lastly applied our protocol to double FISH for the simultaneous detection of miRNAs and cell-specific marker mRNAs. As described for *in situ* hybridization in the Stepwise Procedures, fluorescein-labeled probes for PV, a GABAergic interneuron marker, were hybridized in combination with a DIG-labeled LNA probe for miR-34a. Subsequently, we detected each probe using the TSA system in series. As shown in Figure 7, miR-34a signals were co-localized with PV signals in the TRN, the globus pallidus and the entopeduncular nucleus, where GABAergic neurons are localized (Hernández et al., 2015). ISH using the sense probe of PV mRNA did not detect any ISH signals (data not shown). A genetically targeted miRNA tagging methodology has previously shown that miR-34a is predominantly expressed in PV-positive neurons (He et al., 2012). Consistent with that report, our protocol revealed that miR-34a was expressed in PV-positive neurons of the mouse brain. These results confirm that our experimental conditions can be utilized for double FISH.

Double FISH for the simultaneous detection of miRNAs and mRNAs also yields quantitative information about the expression of miRNAs. For example, the proportion of the miR-34a-expressing cells to total GABAergic neurons in the TRN is able to be quantified. In fact, we counted the miR-34a- and PV-positive cells in the TRN of the section stained by double FISH, and found that ~85% of PV-positive neurons in the TRN were miR-34a-expressing cells (PV positive, 276 cells; both PV and miR-34a positive, 235 cells). Thus, double FISH with a variety of cell-type specific markers would help to expand our understanding about the characterization of miRNA expression.

## Pitfalls

1. Sample preparation: Brain blocks frozen brain by immersion in isopentane chilled with dry ice can be stored at −80°C for at least 1 year. Moreover, sectioned samples can be stored at −80°C for at least a few months.
2. Prehybridization: although we usually perform this step for 30 min, the incubation time of this step can be extended for up to an hour.
3. Hybridization: Like the prehybridization step, the reaction time of this step can be extended for up to 1 day. Attention must be paid to the samples, however, to prevent them from drying out.
4. Post PFA fixation with NBT/BCIP at day 3 is carried out for long-term storage, thus this step can be skipped.

## Artifacts and troubleshooting

1. An acetylation step was performed to reduce the background binding of the negatively charged probe to the tissue sections.
2. Milk casein, a major component in the Blocking Reagent, dissolves well in maleic acid buffer. Therefore, washing with maleic acid buffer was performed to clean up excess casein, along with excess antibodies.
3. Adequate mixing leads to uniform staining when covering the sections with pieces of parafilm during prehybridization and hybridization.
4. The concentration of Proteinase K used and the treatment time applied can be modified.
5. The hybridization temperature should be optimized for each probe.
6. Adding 1-ethyl-3-(3-dimethylaminopropyl) carbodiimide to the PFA fixation solution may help to increase the sensitivity of miRNA ISH.
  IF there is no signal, the following points should be considered.
7. If the threshold cycle value of a miRNA in the punched out tissues is greater than 28 in RT-PCR experiments, it may be difficult to detect the ISH signals of the miRNA.
8. Since the number and position of the replacement LNA of RNA critically affects the sensitivity of ISH signal detection, probes with different position of the replacement LNA of RNA may detect specific signals, although detailed information is not provided by Exiqon.
9. In the case of FISH, the use of an incorrect fluorescence filter will result in inadequate signal separation and produce false positive results.
10. (Optional) In cases where miRNA ISH is combined with immunohistochemistry, proteinase K treatment during ISH may hinder the immunostaining due to antigen digestion.

## Discussion

In this study, we detected low-abundance miRNAs when hybridization was performed at low-stringency and washing at high-stringency, as opposed to hybridizing at high-stringency and washing at a similar or lower-stringency. In several protocol papers (Zimmerman et al., 2013), hybridization was performed using a high-stringency buffer, including 5 × SSC, at no <25°C below its *T*_*m*_-value, and low-stringency washing was performed. High-stringency hybridization conditions are widely used to obtain greater specificity. We detected the signal specific to miR-34a, even with a relatively low-stringency buffer, 2 × SSC, at 37°C below its *T*_*m*_-value. The sodium concentration of 2 × SSC and 5 × SSC are 0.39 and 0.975 M, respectively. According to the nearest neighbor method, a common technique for estimating the *T*_*m*_-value, the different sodium concentrations among these protocols corresponds to a 6.6°C difference in *T*_*m*_-values. Therefore, the hybridization conditions in our protocol have a relatively lower stringency than in previously reported protocols. Since low hybridization stringency can lead to false positive signals, the experimental conditions for each probe should be examined, paying careful attention to stringency. Our data, however, suggest that the detectability of miRNA ISH can be improved by adjusting the balance of stringencies for hybridization and washing.

Recent studies have linked miR-34a to impaired brain function. For instance, miR-34a expression is upregulated in patients with bipolar disorder, and the 25 predicted targets of miR-34a, including the voltage-dependent L-type calcium channel subunits *CACNB3* and *CACNA1C*, overlap with the 140 risk genes identified in genome-wide association studies of bipolar disorder (Bavamian et al., 2015). Additionally, miRNA expression profiling revealed that miR-34a expression is upregulated in patients with schizophrenia (Kim et al., 2010). miR-34a also regulates *SHANK3*, which encodes synaptic scaffolding proteins associated with autism spectrum disorders (Durand et al., 2007). In addition, we showed that miR-34a is expressed in the interneurons of subcortical regions. Since, the entopeduncular nucleus projects to the lateral habenula neuronal subset, thus by innervating aversion-encoding midbrain GABA neurons (Meye et al., 2016), miR-34a could regulate habenula activity via a balance between GABA and glutamate. Thus, *in vivo* analysis of miRNA localization provides reliable and easily interpretable information.

In general, altered miRNA expression has been linked to psychiatric disorders, such as schizophrenia [Baudry et al., 2010; Schizophrenia Psychiatric Genome-Wide Association Study (GWAS) Consortium, 2011]. MiRNA-mediated epigenetic regulation of disease risk genes may be a crucial mechanism in psychiatric diseases. Human genetic and gene targeting studies undertaken by us and others have suggested that pituitary adenylate cyclase-activating polypeptide (PACAP) is a risk factor for psychiatric disorders, such as schizophrenia (Hashimoto et al., 2001, 2007; Ressler et al., 2011; Hazama et al., 2014). In addition, our previous ISH studies show *in situ* localization of PACAP-receptor type1 in the brain with PAC1 ISH (Hashimoto et al., 1996), and abnormalities in photic entrainments in PACAP-deficient mice (Kawaguchi et al., 2010). Thus, *in situ* detection of miRNAs, in addition to mRNAs, would provide direction for future functional studies.

In summary, we focused on sample fixation, probe accessibility and stringency in our protocol for nucleic acid hybridization for miRNAs. We successfully detected low-abundance miRNAs, including miR-34a, miR-34c, miR-181a, miR-100, and miR-219, using LNA probes and identified miRNA-expressing cells using a double FISH technique in brain tissues. Moreover, our results revealed that a low hybridization temperature followed by high stringency washes might be better than previous methods to detect miRNA signals by ISH. This method can lower the technical hurdles in the study of spatial expression patterns of miRNAs and the detailed roles of miRNAs in brain function.

## Author contributions

AK and HH designed the research and wrote the paper. TN designed the research protocols. SK, HM, RO, and KH performed experiments. AH, MN, and NS analyzed data.

## Funding

This work was supported in part by the Japan Society for the Promotion of Science (JSPS) Grants-in-Aid for Scientific Research, Grant Numbers JP25670037 (AK), JP15K14964 (AK), JP26293020 (HH), JP26670122 (HH), JP15H01288 (HH); the Takeda Science Foundation, Japan (AK); the Funding Program for Next Generation World-Leading Researchers, Grant No. LS081 (HH); the Uehara Memorial Foundation, Japan (HH); the JSPS Program for Advancing Strategic International Networks to Accelerate the Circulation of Talented Researchers, Grant No. S2603 (HH); and the Strategic Research Program for Brain Sciences from Japan Agency for Medical Research and Development (HH).

### Conflict of interest statement

The authors declare that the research was conducted in the absence of any commercial or financial relationships that could be construed as a potential conflict of interest.
